# Is inpatient rehabilitation a predictor of a lower incidence of persistent knee pain 3-months following total knee replacement? A retrospective, observational study

**DOI:** 10.1186/s12891-022-05800-0

**Published:** 2022-09-12

**Authors:** Nathan Johns, Justine Naylor, Dean McKenzie, Bernadette Brady, John Olver

**Affiliations:** 1 Epworth Monash Rehabilitation Medicine Unit. Suite 2.4, 32 Erin Street, Richmond, VIC 3121 Australia; 2 Whitlam Orthopaedic Research Centre, Orthopaedic Department, Liverpool Hospital, Locked Bag 7103, Liverpool BC, NSW 1871 Sydney, Australia; 3 Research Development and Governance Unit, Epworth HealthCare, 89 Bridge Road, Richmond, VIC 3121 Australia; 4 Department of Epidemiology and Preventive Medicine, Monash University, 553 St Kilda Road, Melbourne, VIC 3004 Australia; 5 Physiotherapy Department & Department of Pain Medicine, Liverpool Hospital, Locked Bag 7103 Liverpool BC, Sydney, NSW 1871 Australia

**Keywords:** Chronic pain, Knee arthroplasty, Rehabilitation, Osteoarthritis, Chronic Knee pain

## Abstract

**Background:**

Moderate to severe levels of persistent knee pain have been estimated to affect up to 25% of people 3-months or more after a total knee replacement. It is unknown whether the type of rehabilitation pathway is associated with persistent high pain after surgery. Using a prospectively followed Australian cohort who underwent total knee replacement for knee osteoarthritis, this study aimed to i) report the incidence of high-intensity knee pain (defined as a score ≤ 15 on the Oxford Knee Score pain subscale) across time and ii) identify whether referral to inpatient rehabilitation was one of the predictors of persistent pain at 3-months post-surgery.

**Methods:**

A retrospective analysis of a large prospective study was conducted using the Oxford Knee Score pain subscale to determine if participants had high pain at 3-months, 12-months and 36-months post-surgery. Relative risks for high pain at 3-, 12- and 36-months between the type of rehabilitation pathway were determined using Poisson multivariable regression with robust standard errors. The same technique was also employed to determine potential predictors, including rehabilitation pathway, of high pain at 3 months.

**Results:**

The incidence of high pain in all participants was 73% pre-surgery and 10, 5 and 6% at 3-, 12- and 36-months respectively following knee replacement. There was a significant interaction between time and rehabilitation pathway, suggesting that the effect of the rehabilitation pathway varied across time. The incidence of high pain at 3-months did not significantly differ between those who attended inpatient rehabilitation (11.6%) and those discharged directly home (9.5%). Multivariable Poisson regression analysis identified the pre-surgical presence of high pain, co-morbid low back pain or other lower limb problem, younger age and having a major complication within 3-months following surgery as significant predictors of persistent pain whilst discharge to inpatient rehabilitation was not.

**Conclusion:**

A small but clinically significant minority of people continued to have high pain levels at 3-, 12- and 36-months following a primary total knee replacement for osteoarthritis. Participation in an inpatient rehabilitation program does not appear to be an important predictor of ongoing knee pain.

**Trial registration:**

The data were collected in the Evidence-based Processes and Outcomes of Care (EPOC) study, ClinicalTrials.gov Identifier: NCT01899443

**Supplementary Information:**

The online version contains supplementary material available at 10.1186/s12891-022-05800-0.

## Background

Moderate to severe persistent knee pain, defined as pain persisting beyond 3 post-operative months following a primary total knee replacement, has been estimated in a systematic review of observational studies to affect between 8 to 26% of people [1]. The Australian Orthopaedic National Joint Replacement Registry reported that 8% of people had severe pain and 17.4% had moderate pain at 6-months post-knee replacement [2]. A European observational study of 63 knee replacement recipients found that at 12-months, 8% of patients reported severe pain, 19% moderate pain and 38% reported mild pain [3].

Pain persisting after a knee replacement is associated with reduced levels of patient satisfaction [4] and reduced quality of life [5]. Persistent (or chronic) pain itself is associated with high years of life lost to disability and high community and personal financial costs [6]. For these reasons, identifying a preventative treatment for persistent knee pain is a worthwhile endeavour.

There are known pre-operative risk factors that are associated with persistent pain following a total knee replacement. Symptom and symptom management factors include higher pain intensity [7, 8], higher pain interference with walking [9, 10], widespread pain [7, 8, 11–14], sensitization [8, 15, 16] and poor coping strategies [16]; co-morbid factors include obesity [17] and the presence of low back pain [18]; psychological factors [19–21] such as depression [7, 22], anxiety [8, 10, 22, 23] and catastrophizing [7, 13, 16, 24] and social factors such as higher educational levels [10]. A systematic review of post-operative risk factors concluded that there was insufficient evidence of an association of persistent pain at 6-months post-surgery and early post-operative pain levels, function or psychosocial factors [25].

The effect of different rehabilitation approaches on either the prevention or incidence of persistent pain following knee replacement has received little attention in the literature. This is despite approximately 25% of people in Australia being referred to inpatient rehabilitation [26, 27] and some form of rehabilitation being almost universally provided in the early subacute period after surgery. Several studies have concluded that there appears to be no superiority of one rehabilitation mode over another for *average pain* and function outcomes early [28], at 6-months [29] or at one year post-surgery [30], but it is unclear whether there is a difference in the proportion of people reporting *high pain* at various time periods. Given some modes of rehabilitation (e.g inpatient rehabilitation) are vastly more costly than other modes (e.g home-based programs) [31], it would be reasonable to expect that the incidence of persistent high pain longer-term may be lower with the more resource-intensive modes, if indeed this outcome is amenable to rehabilitation.

The aim of this study was: i) to determine the incidence of high-level knee pain across time following total knee replacement and ii) to determine whether the incidence varied according to participation in an inpatient rehabilitation program or not at 3-months post-surgery.

## Patients and methods

### Study type

This study constitutes a retrospective analysis of anonymised data previously collected in EPOC (Evidenced-based Processes and Outcomes of Care), a large multi-centre prospective observational study of people recruited between August 2013 and January 2015 and a 3-year follow-up continuing until 2018 (ClinicalTrials.gov Identifier: NCT01899443). Ethical approvals for the original studies [31–34] were provided by multiple ethics committees and are detailed under ‘Declarations’.

### Participants

The EPOC study enrolled participants pre-operatively between August 2013 to January 2015 from 19 private and public hospitals from 5 states in Australia where high volumes of joint replacement were performed. The larger EPOC study evaluated participants having either a primary knee or hip replacement, however, this paper only evaluated those receiving a knee replacement. At pre-operative assessments, patients were screened and recruited by trained site co-ordinators. Participants were eligible if they were over 18 years of age, had osteoarthritis recorded as their primary diagnosis in the index knee, were undergoing an elective primary total knee replacement and were able to understand English. Participants were excluded if they had cognitive impairment or a history of dementia, were undergoing a revision knee replacement, having a knee replacement for a condition other than osteoarthritis, were unable to understand English, under 18 years of age or were planning a second joint replacement within 3-months.

### Intervention

For the purpose of this study, participants were divided into 2 groups dependent on their post-operative rehabilitation pathway - discharged directly from the acute ward to Inpatient Rehabilitation (IR) or discharged directly home (Home Group). The Inpatient Rehabilitation group included participants who were discharged to inpatient rehabilitation only as well as those participants who were discharged to inpatient rehabilitation and subsequently received outpatient therapy once discharged. Inpatient rehabilitation in Australia generally involves a structured goal-based program of almost daily physiotherapy combined with occupational therapy, medical care by Rehabilitation Physicians, rehabilitation nursing care and care from other members of an interdisciplinary team as required. Outpatient therapy included public or private physiotherapy sessions or day hospital attendance each of which could include hydrotherapy. The Home Group included participants who were discharged directly home from the acute ward and received either outpatient therapy (as above), a monitored home program supervised by up to 3 physiotherapist visits and those who had an unmonitored home program (no supervised sessions). The duration and frequency of outpatient therapy has been published elsewhere [31].

### Variables

Baseline data were collected pre-operatively and further telephone assessments were conducted at 3-months, 12-months and 36-months post-operatively. Baseline data were collected on age; body mass index (BMI); sex; private health status; educational level; financial status; hospital (private or public); analgesia being taken; the presence of comorbidities including a heart problem, lung disease, diabetes, a history of cancer, a neurological disorder, Parkinson’s disease, stroke, hypertension, hypercholesterolemia, liver disease, hepatitis, kidney disease, bleeding disorder, obstructive sleep apnoea, gastroesophageal reflux disease; a previous hip or knee replacement; the presence of low back pain or other lower limb problem; depression; anxiety and smoking status. Patient-reported surveys were also collected including the EuroQol (EQ-5D-5L) [35] survey and the Oxford Knee Score (OKS) [36]. The OKS was used to calculate the primary outcome and was collected pre-surgery and then at 3-, 12- and 36-months post knee replacement. The Oxford Knee Score is a 12-item patient-reported outcome measure, that can be divided into pain and functional subscales [37]. Patients rate items based on their experience over the prior 4 weeks using a 5-point Likert scale which is then converted into a score for each item, where 4 is the best score (for example, *no* knee pain) and 0 is the worst score (for example *severe* knee pain) [38]. The pain subscale incorporates 7 items with the remaining 5 items corresponding to the functional subscale. The lowest score of the pain subscale is 0 (severe pain or severe pain interference in every category) and the highest score is 28 (no pain or no pain interference in every category). This pain subscale has high sensitivity and specificity for identifying a “high pain” group who have total scores of less than 15 (range 0 to 14) which is associated with reduced function and reduced quality of life [37]. Alongside the patient reported measures, the American College of Anaesthesiologists’ (ASA) score [39] was also recorded at the time of operation. Similarly, acute complications were also recorded. At 3-months post-surgery, patients were followed-up by telephone; patient-reported outcomes and complications experienced were obtained. Major complications occurring within 3-months included deep venous thrombosis (DVT), pulmonary embolus (PE), wound dehiscence or superficial infection, deep wound infection, manipulation under anaesthetic, acute myocardial infarction, ischemic heart disease, congestive cardiac failure, stroke, pneumonia or atelectasis. Telephone follow-up continued at 12 and 36 months for those available.

### Primary outcome

The incidence and relative risks of high pain (an Oxford Knee Score Pain Subscale score of 0 to 14) at 3-months post-surgery by inpatient rehabilitation or home group.

### Statistical analysis

The sample size was dictated by the primary study, thus no a priori sample size calculation was performed. Oxford Knee Scores were converted into pain subscale scores to determine high pain (subscale score 0–14) and not high pain (subscale score of 15–28). To determine the relative risk (RR) of having high pain across time points, Poisson regression with robust standard errors [40–42] was used, to examine the overall or main effect of time baseline, 3, 12, 36 months), group (Inpatient Rehabilitation or Home) and any statistical interaction between them [43], for example, evaluating the difference between the two groups across time. To determine the incidence of high pain in the Inpatient Rehabilitation and Home groups, proportions were determined and risk ratios calculated using the clinically relevant potential predictors of persistent high pain, outlined above. These potential predictors, including rehabilitation pathway, were examined individually in separate Poisson regressions, as well as jointly in a multivariable Poisson regression.

For comparison of differences between those who were and were not retained at follow-up, as well as Inpatient Rehabilitation and Home groups, Poisson regression with robust standard errors was also employed to compare categorical outcomes, while independent samples t-tests were performed for continuous variables (Supplement 1). Alpha was set to 0.05, 2-tailed. 95% confidence intervals (CI) (Clopper-Pearson exact binomial in the case of binary variables [44]) were reported throughout. All analyses were performed using Stata 17 (Stata Corporation LLC, College Station, Texas, USA, 2021).

## Results

**Fig. 1:**
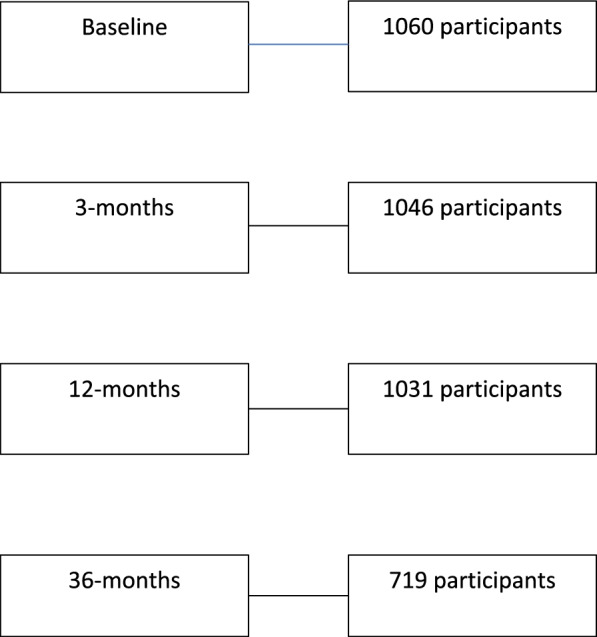
Retention of participants from baseline to 36-months

From the baseline (pre-surgery) sample of 1060 participants, a total of 375 (35.4%) were discharged to Inpatient Rehabilitation and 685 (64.6%) were discharged directly Home. There were statistically significant differences between the groups at baseline and these are documented in Supplement 2. The median inpatient rehabilitation length of stay was 13 days (25% percentile 8 days, 75% percentile 14 days). Forty-eight per cent of total participants were recruited from private hospitals, 55.7% were female, the average age was 68.4 years (standard deviation (SD) = 8.7 years) and the mean BMI was 32.1 kg/m^2^ (SD = 6.7 kg/m^2^). The retention of participants over 36-months is illustrated in Fig. 1. Cohort retention was high up to 12-months and 67% remained available for follow-up at 36-months. Equal numbers of patients with high pain at 3- and 12-months were retained (summarised in Supplement 1). Patients lost to follow-up at 36-months were more likely to have reported higher pain (relative risk RR = 1.09, 95% CI 1.01–1.17, *p* = 0.03) and lower OKS functional scale (mean difference = 0.7, 95% CI 0.3–1.2, *p* = 0.004) at baseline and less likely to have been in inpatient rehabilitation (RR = 0.75, 95% CI 0.62–0.90, *p* = 0.003) than those retained.

### High pain pre-surgery and at 3-months, 12-months and 36-months post-surgery

Pre-surgery, 776 of 1060 (73.2%) of participants enrolled to have a primary total knee replacement had high pain (Table 1). The incidence of high pain at 3-months post-surgery was significantly reduced to 10.2% (107 of 1046 participants) compared with pre-surgery (relative risk 0.14, 95% CI 0.12–0.17, *p* < 0.001). The incidence of high pain at 12-months was 4.8% (50 of the 1031 participants) and 6.5% (47 of the 719 participants) at 36-months.

**Table 1:** High pain by discharge destination pre-surgery to 36-months

	Total High Pain/total (percent)	Inpatient rehab High Pain/total (percent)	Home High Pain/total (percent)	Relative Risk (RR)
Time point				
Pre-surgery	776/1060 (73.2%)	257/375 (68.5%)	519/685 (75.8%)	RR = 0.90 CI 0.83–0.98 ***p*** = 0.015*
3-months	107/1046 (10.2%)	43/371 (11.6%)	64 /675 (9.5%)	RR = 1.22 CI 0.85–1.76 *p* = 0.28
12-months	50/1031 (4.8%)	15/364 (4.1%)	35/667 (5.2%)	RR = 0.79 CI 0.43–1.42 *p* = 0.42
36-months	47/719 (6.5%)	12/278 (4.3%)	35/ 441 (7.9%)	RR = 0.54 CI 0.29–1.03 *p* = 0.06

Legend: *RR* relative risk, *CI* 95% confidence interval, *p p*-value

### Comparing the risk of high pain between the inpatient rehabilitation and home groups

Pre-surgery, the percentage of participants with high pain was significantly lower in the group who would later go to Inpatient Rehabilitation (67.6%) compared with those who would be discharged directly home (75.1%, relative risk = 0.90, 95% CI 0.83–0.98, *p* = 0.01) (Table 1). At 3-months following knee replacement, there were more participants with high pain in the Inpatient Rehabilitation group (11.6%) compared with the Home group (9.5%) however, this was not statistically significant (relative risk = 1.22, 95% CI 0.85–1.76, *p* = 0.28). At 12-months, there was a lower incidence of high pain in the Inpatient Rehabilitation group but this clearly did not reach statistical significance (relative risk = 0.79, CI 0.43–1.42, *p* = 0.42). Again, at 36-months, there was a lower risk of high pain in the Inpatient Rehabilitation group that did not reach statistical significance (relative risk = 0.54, CI 0.29–1.03, *p* = 0.06). A test of the statistical interaction between timepoint and Inpatient Rehabilitation and Home groups, indicated that the interaction was marginally statistically significant (likelihood ratio chi-square (3) = 8.0, *p* = 0.045); in other words, the magnitude of the relative risks for high pain between the two groups significantly differed across time.

### Adjusted relative risk of high pain at 3-months post-surgery

The results from the multivariable Poisson regression analysis performed to look for potential predictor variables of high pain at 3-months are seen in Table 2.

**Table 2:** Multivariable Poisson regression analysis for high pain at 3-months post-surgery

Variable	Adjusted Relative risk	95% confidence interval	*P* - value
Inpatient rehab or home	1.32	0.92–1.89	0.14
Age	0.95	0.93–0.97	< 0.001*
BMI	0.99	0.97–1.01	0.38
Smoker	1.48	0.91–2.40	0.12
OKS function subscale baseline	0.95	0.89–1.01	0.13
High pain baseline	2.18	1.03–4.64	0.043*
Opioids baseline	1.24	0.82–1.87	0.31
Gender (female)	1.07	0.74-1.54	0.74
Low back pain or other lower limb problem	1.64	1.11–2.42	0.013*
Major complication	1.99	1.40–2.83	< 0.001*
ASA category	1.25	0.85–1.83	0.26
Depression or anxiety	1.10	0.73–1.64	0.65
EQVAS baseline	1.00	0.99–1.01	0.82

Legend: *BMI* Body Mass Index (Kg/m^2^), *OKS* Oxford Knee Score, *ASA* American College of Anaesthesiologists, *EQVAS* EuroQol Visual Analogue Scale

Inpatient rehabilitation compared to discharge home following knee replacement was not significantly associated with high pain at 3-months (adjusted relative risk 1.32, 95% CI 0.92–1.89, *p* = 0.14). A younger age (relative risk 0.95, 95% CI 0.93–0.97, *p* < 0.001), high pain pre-surgery (relative risk 2.18, 95% CI 1.03–4.64, *p* = 0.043), low back or other lower limb problem pre-surgery (relative risk 1.64, 95% CI 1.11–2.42, *p* = 0.013) and a major complication occurring within the first 3 months (relative risk 1.99, 95% CI 1.40–2.83, *p* < 0.001) were all associated with high pain at 3-months.

## Discussion

This study reports the trajectories of high index joint pain in a large Australian sample of people followed prospectively after a primary knee replacement for osteoarthritis. Approximately 10% of participants had high pain at 3-months, 5% at 12-months and 6.5% at 36-months post-surgery. These values are at the lower end of percentages in other studies and in registries (1–3). High pain was found to be associated with lower age, high pain pre-surgery, the presence of pre-surgical back or other lower limb problem and having a major complication within the first 3-months. Inpatient Rehabilitation was not associated with high pain following knee replacement compared with the Home Group at any time point. More specifically, it was not associated with high pain at 3, 12 and 36 months whether or not adjustment for potential confounders was undertaken.

It could be considered surprising that inpatient rehabilitation was not associated with a reduced incidence of persistent pain, given its multidisciplinary nature and focus on reduction of pain and distress [45]. The relatively high level of staffing for inpatient rehabilitation, which may include psychologists, provides an opportunity to address the multi-dimensional nature of pain and its risk factors such as catastrophizing, stress, anxiety, depression and coping strategies. There should exist the opportunity to provide pain neuroscience education [46, 47], relaxation strategies and sleep strategies as well as a more personalised approach to pain reduction than during limited outpatient sessions. Multi-disciplinary biopsychosocial outpatient programs are effective in reducing pain and disability in persistent musculoskeletal pain [48]. But we can only speculate as to why we found no difference in pain outcomes or the extent to which inpatient rehabilitation included pain-specific approaches.. It is possible that high pain is related to factors not amenable to rehabilitation treatments (such as high pre-surgical pain or complication) or that the currently available programs typically provided are not tailored to address or prevent persistent pain [49]. It is possible that the exercise component of the rehabilitation program may not have differed significantly between the groups in terms of amount or intensity.

Systematic reviews of post-operative treatments after knee replacement have only identified small effects on pain. In one systematic review of physiotherapy commencing immediately following a total knee replacement, there was evidence for only a small mean between-group difference in pain (measured using patient-reported outcome measures) at 3–4 months when compared to no therapy [29]. Another systematic review [50] evaluating the effect of post-hospital discharge rehabilitation and physiotherapy interventions delivered within the first 3 months post-knee replacement found that only a single study of home-based functional exercises designed to reduce kinesiophobia was effective at reducing long term pain [51]. Thus, it is possible that targeted treatments, focusing on cognitive as well as physical rehabilitation, are important for the treatment and prevention of persistent pain.

Given the low frequency of high pain from 3-months on and that a strong predictor is a post-operative complication, it may be difficult to *identify* those patients pre-operatively who need a targeted pain rehabilitation program to prevent high pain.

Combined, knee replacement surgery and the subsequent treatments appear to be largely successful in reducing the burden of high pain from over 70% of this sampled population to about 10% by 3-months. This result compares favourably to two UK studies using the same methodology, one finding that 30% of the sample had high pain at 10 weeks [52]. The second study examined data from the British National Joint Registry and identified 15% of people with high pain 6-months following knee replacement [53]. This notwithstanding, even with a seemingly low incidence of high pain of 5% at 12-months, this equates to an extra 2750 people a year in Australia living with high pain post-knee replacement. With the volume expected to increase to 160,000 primary knee replacements annually by 2030 [54], we can anticipate an additional 8000 people a year with high pain 12-months following a knee replacement.

This study has several strengths. The data were collected prospectively and minimal loss to follow-up was evident at 3- and 12-months. The population profile is similar to that commonly reported in the Australian National Joint Replacement Registry supporting its generalisability [2]. An extensive list of covariates (both from the patients and from the medical record) were captured enabling inclusion in the prediction modelling. It is also the first study to our knowledge inclusive of the rehabilitation pathway as a potential predictor of persistent pain after knee replacement, thus it extends the extant literature concerning the predictors of persistent pain. Limitations include the study design (observational as opposed to randomised) and the loss to follow-up at 36-months. The greater loss to follow-up at 36 months of those with high pain at baseline and less likely to be referred to inpatient rehabilitation potentially renders it less likely for our results to identify referral to inpatient rehabilitation as protective of high pain at this time. However, as IR was not protective at the earlier time points, it is considered unilikely that it is protective at 36 months. Finally, it is unknown whether patients received other treatments for pain after the conclusion of formalised rehabilitation so it is unknown whether any treatments modified the association between rehabilitation pathway and high pain.

## Conclusions

The incidence of high levels of pain after total knee replacement are comparatively low 3-months post-surgery compared to pre-surgery levels, and the incidence continues to decline thereafter. Several patient-level factors predict the presence of high pain at 3 months post-surgery; referral to inpatient rehabilitation does not appear to be an important factor. Whilst the incidence of persistent pain is relatively low, the impact of pain on these individuals is likely to be clinically significant and thus is worthy of prevention if possible.

## Supplementary Information

**Additional file 1.**

## Data Availability

The data that support the findings of this study are held in a data repository at Monash University “Bridges”. The DOI is 10.26180/19709701 and will be made public upon publication of this manuscript. The data can be made available to reviewers and editors as requested.
