# Supplementary material for: Is inpatient rehabilitation a predictor of a lower incidence of persistent knee pain 3-months following total knee replacement? A retrospective, observational study
Source: BMC Musculoskelet Disord. 2022 Sep 12;23:855. doi: 10.1186/s12891-022-05800-0 (PMC9465848; doi:10.1186/s12891-022-05800-0)
Supplement: Supplementary file 1 — Additional file 1. [file 12891_2022_5800_MOESM1_ESM.docx]

Supplements:

Supplement 1: Characteristics of participants retained versus lost at 36 months follow-up

| Variable | Retained | Lost at 36-months | statistic |
| --- | --- | --- | --- |
| Sex (female) | 415/722  (57.5%) | 186/357  (52.1%) | RR =0,90  95% CI 0.81 - 1.02  p = 0.10 |
| Age (years) -mean | 68.4  SD 8.5 | 68.5  SD 9.2 | Diff. = -0.1  95% CI: -1.2 – 1.0  t = -0.11  p = 0.91 |
| BMI (Kg/ m^2^) -mean | 32.14  SD 6.5 | 32.15  SD 6.9 | Diff. = -0.01  95% CI: -0.86 – 0.83  t= -0.03  p = 0.97 |
| High Pain at baseline | 510/715  (71.3%) | 273/352  (77.6%) | RR = 1.09  95% CI 1.01- 1.17  **p = 0.03** |
| High Pain at 3-months | 74/722  (10.3%) | 33/329  (10.0%) | RR = 0.97  95% CI 0.66 – 1.44  p= 0.91 |
| High Pain at 12-months | 34/721  (4.7%) | 16/315  (5.1%) | RR = 1.08  95% CI 0.60 -1.92  p = 0.80 |
| Baseline OKS functional scale | Mean = 10.4  SD 3.7 | Mean = 9.7  SD 3.6 | Diff = 0.7  95% CI: 0.2 – 1.2  t = 2.9  **p = 0.004** |
| Inpatient rehabilitation | 278/719  (38.7%) | 102/352  (29.0%) | RR = 0.75  95% CI 0.62 – 0.90  **p = 0.003** |
| Major complication within the first 3-months | 178/721  (24.7%) | 89/ 338  (26.3%) | RR = 1.06  95% CI 0.86 – 1.33  p = 0.57 |
| Back pain or other lower limb problem | 334/721  (46.3%) | 162/ 356  (45.5%) | RR = 0.99  95% CI 0.95 – 1.04  p = 0.80 |

Legend: RR = relative risk; 95% CI = 95% confidence interval; SD = standard deviation, p = p-value; BMI = Body Mass Index; OKS = Oxford Knee Score

Supplement 2: Differences in variables between Inpatient Rehabilitation (IR) and Home groups

| Variable | Total number (percent) | IR (380) | Home (691) | Significance |
| --- | --- | --- | --- | --- |
| Sex (female) | 595 (55.7%) | 234 (61.6%) | 361 (52.2%) | RR = 0.78  CI 0.66 – 0.92  **p=0.004*** |
| Age (years) | Mean 68.4  SD 8.7  (range 37-91) | Mean 69.6  SD 8.6  CI 68.7 - 70.4 | Mean 67.7  SD 8.7  CI 67.1 – 68.4 | Diff = 1.9  CI 0.7-2.9  t = 3.3  **p=0.001*** |
| BMI (Kg/m^2^) | Mean 32.1  SD 6.7  (range:18.5-64.8) | Mean 31.9  SD 6.8  CI 31.2 – 32.6 | Mean 32.3  SD 6.5  CI 31.8 - 32.7 | Diff = -0.4  CI -1.2 – 0.4  T =0.9  p= 0.36 |
| Diabetes (N, %) | 218 (20.7%) | 69 (18.2%) | 155 (22.4%) | RR= 0.81  CI 0.63-1.04  p=0.1 |
| Smoking status   - Never/ Ex - Current | 985 (92.6%)  79 (7.4%) | 360 (95.2%)  18 (4.8%) | 625 (91.1%)  61 (8.9%) | RR= 0.62  95%CI 0.41 -0.94  **p=0.025** |
| Previous TKR | 211 (21.4%) | 66 (18.9%) | 145 (22.8%) | RR = 0.86  CI 0.70-1.06  p=0.16 |
| Baseline OKS functional subscale | Mean 10.1  SD 3.7 | Mean 10.3  SD 3.7  CI 9.9-10.7 | Mean 10.1  SD 3.6  CI 9.8-10.4 | Diff = 0.2  CI -0.2 to 0.7  t = 1.05,  p=0.3 |
| Baseline OKS  pain subscale | Mean 11.4  SD 5.2 | Mean 12.0  SD 5.3  CI 11.5-12.5 | Mean 11.2  SD 5.1  CI 10.8-11.5 | Diff = 0.8  CI -0.2 to 1.5  t = 2.5  **p=0.01*** |
| High pain at baseline (N, %) | 776 (73.2%) | 257 (68.5%) | 519 (75.8%) | RR = 0.90  CI 0.83-0.98  **p= 0.01*** |
| Surgery at private hospital (N, %) | 510 (47.6%) | 310 (81.6%) | 200 (28.9%) | RR = 2.8  CI 2.5-3.2  **p<0.001*** |
| Opioid taken at baseline  (N, %) | 167 (15.6%) | 56 (14.7%) | 111 (16.1%) | RR =0.92  CI 0.68-1.23  p=0.6 |
| EQVAS# at baseline | Mean 71.4  SD 0.56  CI 70.3 – 72.5 | Mean 69.7  SD 18.6  CI 70.3-72.5 | Mean 72.3  SD 17.9  CI 71.0 -73.7 | Diff = - 2.6  CI -4.9 to – 0.37  t=2.3  **p=0.02*** |
| Educational level   1. Year 10 or below 2. Completed/ TAFE 3. University | 436 (40.5%)  453 (42.1%)  188 (17.4%) | 116 (30.7%)  154 (40.7%)  108 (28.6%) | 316 (45.7%)  297 (43%)  78 (11.3%) | RR = 1  RR=1.27, CI 1.04 -1.56, **p=0.02***  RR=2.16, CI 1.77-2.64, **p<0.001*** |
| Any comorbidity | 983 (93.4%) | 362 (95.3%) | 638 (92.3%) | RR = 1.03  CI 1.0-1.06  p= 0.07 |
| ASA Score   - 1 & 2 - 3 & 4 | 660 (63.6%)  378 (36.4%) | 218 (58.8%)  153 (41.2%) | 442 (66.3%) 225 (33.7%) | RR =1  RR = 1.23  95% CI 1.04 - 1.44  **p=0.015*** |
| Low back pain or other lower limb problem | 483 (46.0%) | 187 (49.3%) | 304 (44.0%) | RR = 1.12  CI 0.98-1.28  p=0.10 |
| Combined history of depression or anxiety | 221 (20.6%) | 69 (18.2%) | 152 (22%) | RR = 0.85  CI 0.69 – 1.06  p=0.15 |
| Major complication within the first 3-months | 261 (24.4%) | 91 (24%) | 170 (24.6%) | RR = 0.96  CI 0.80 – 1.14  p=0.63 |

Legend: SE = standard error, SD = standard deviation, CI = 95% confidence interval, p = p-value, RR = Relative Risk, TAFE = Technical and Further Education, ASA = American Society of Anesthesiologists

* Statistically significant, p-value <0.05

^#^The EQ-5D-5L is a descriptive patient reported outcome measure covering the domains of mobility, pain/ discomfort, self-care, anxiety/ depression and usual activities (49). It also encompasses a visual analogue scale of self-rated overall health (EQ-VAS) scored from 0 to 100, where 100 is the best score.

Supplement 3: Association of variables with high pain at 3 months

| Variable | Total  Number (percent) | High Pain | Not High Pain | Statistics |
| --- | --- | --- | --- | --- |
| Sex (female) | 588 (55.7 %) | 67 (62.6%) | 521 (55.2%) | RR = 0.76  CI 0.52-1.10  P=0.15 |
| Age (years) | Mean 68.4 (range 37-91) | Mean 64.2  CI 62.5-65.9 | Mean 68.9  CI 68.3-69.4 | RR =0.95  CI 0.93-0.97  P**<0.001*** |
| BMI (kg/m2) | Mean 32.1  SD 6.7 | Mean 33.8  SD 7.5 | Mean 31.9  SD 6.5 | Diff = 1.9  CI 0.54 – 3.20  t=2.77  **P=0.006*** |
| History of Diabetes | 218 (20.7%) | 25 (23.4%) | 193 (20.4%) | RR = 1.16  CI 0.76-1.78  P=0.50 |
| Smoking status   - Never/ Ex - Current | 967 (92.5%)  79 (7.5%) | 92 (86.0%)  15 (14.0%) | 875 (93.2%)  64 (6.8%) | RR = 2.0  95% CI 1.21 -3.28  **P= 0.006*** |
| Previous TKR | 223 (21.2%) | 19 (8.5%) | 88 (10.6%) | RR =0.80  CI 0.50-1.29  P=0.36 |
| Baseline OKS function sub-scale score | Mean 10.1  SE 0.11  CI 9.9-10.4 | Mean 8.6  SE 0.30  CI 8.0 – 9.2 | Mean 10.3  SE 0.12  CI 10.1 – 10.6 | Diff =- 1.7  CI - 1.01 to -2.46  t= - 4.7  **P<0.001*** |
| Baseline OKS pain subscale | Mean 11.4  SE 0.16  CI 11.1-11.7 | Mean 8.6  SE 0.42  CI 7.75 – 9.41 | Mean 11.8  SE 0.17  CI 11.4-12.1 | Diff = -3.2  CI -2.18 to – 4.22  t =-6.1  **P< 0.001*** |
| High Pain at baseline | 764 (73.2%) | 98 (91.6%) | 666 (71.1%) | RR = 3.99  CI 2.04 –7.79  **P<0.001*** |
| Surgery at Private hospital  N, % | 503 (47.9%) | 45 (42.1%) | 458 (48.5%) | RR=1.26  CI 0.88-1.82  P=0.21 |
| Taking opioid (yes) at baseline | 162 (15.4%) | 28 (26.1%) | 134 (14.1%) | RR =1.94  CI 1.31-2.89  **P=0.001 *** |
| EQVAS baseline | Mean 71.4  SE 0.56  CI 70.3 – 72.5 | Mean 65.2  SD 19.3  CI 61.5-68.9 | Mean 72.0  SD 17.8  CI 70.9-73.2 | Diff = -6.8  CI -3.27 to -10.47  t=-3.75  **P<0.001*** |
| Educational level   1. Year 10 or below 2. Completed/ TAFE 3. University | 430 (40.5%)  453 (42.1%)  188 (17.4%) | 36 (33.6%)  58 (54.2%)  13 (12.2%) | 387 (41.1%)  383 (40.6%)  172 (18.3%) | 1  RR = 1.55,  CI 1.04-2.29  **p =0.03**  RR= 0.83,  CI 0.45-1.52  p=0.54 |
| Any Comorbidity | 982 (93.4%) | 98 (91.6%) | 884 (93.6%) | RR =0.77  CI 0.40-1.45  P=0.41 |
| ASA score   - 1 & 2 - 3 & 4 | 646 (63.3%)  375 (36.7%) | 57 (54.8%)  589 (64.2%) | 47 (45.2%)  328 (35.8%) | RR = 1.42  95% CI 0.99 - 2.05  P =0.06 |
| Low back pain or other Lower Limb problem | 483 (46.0%) | 67 (62.6%) | 416 (44.1%) | RR =1.97  CI =1.35 -2.85  **P<0.001*** |
| History of depression or anxiety | 213 (20.3%) | 31 (29.0%) | 182 (19.3%) | RR = 1.60  CI 1.09 – 2.37  **P=0.02** |
| Major complication within first 3-months | 262 (24.9%) | 43 (40.2%) | 219 (23.2%) | RR= 2.03  CI 1.41-2.90  **P<0.001*** |

RR = relative risk, P = p-value, CI = 95% confidence interval, OKS = Oxford Knee Score, BMI = Body Mass Index; ASA = American Society of Anesthesiologists; TAFE = Technical and Further Education; EQVAS = EuroQol Visual Analogue Scale

* statistically significant p-value
